# Use of methylene blue and near-infrared fluorescence in thyroid and parathyroid surgery

**DOI:** 10.1007/s00423-017-1641-2

**Published:** 2017-12-11

**Authors:** Sarah L. Hillary, Stephanie Guillermet, Nicola J. Brown, Sabapathy P. Balasubramanian

**Affiliations:** 1 Sheffield Teaching Hospitals NHS Foundation Trust, c/o Room F25, Royal Hallamshire Hospital, Glossop Road, Sheffield, South Yorkshire S10 2JF UK; 2 Department of Oncology and Metabolism, University of Sheffield, Sheffield, UK; 3 Fluoptics, Grenoble, France

**Keywords:** Methylene blue, Near-infrared, Fluorescence, Localisation, Parathyroid, Intraoperative

## Abstract

**Purpose:**

Intraoperative localisation and preservation of parathyroid glands improves outcomes following thyroid and parathyroid surgery. This can be facilitated by fluorescent imaging and methylene blue; a fluorophore is thought to be taken up avidly by parathyroid glands. This preliminary study aims to identify the optimum dose of methylene blue (MB), fluorescent patterns of thyroid and parathyroid glands and develop a protocol for the use of intravenous MB emitted fluorescence to enable parathyroid identification.

**Methods:**

This is a phase 1b, interventional study (NCT02089542) involving 41 patients undergoing thyroid and/or parathyroid surgery. After exposure of the thyroid and/or parathyroid gland(s), intravenous boluses of between 0.05 and 0.5 mg/kg of MB were injected. Fluobeam® (a hand held fluorescence real-time imager) was used to record fluorescence from the operating field prior and up to 10 min following administration.

**Results:**

The optimum dose of MB to visualise thyroid and parathyroid glands was 0.4 mg/kg body weight. The median time to onset of fluorescence was 23 and 22 s and the median time to peak fluorescence was 41.5 and 40 s, respectively. The peak fluorescence for thyroid and parathyroid glands compared to muscle were 2.6 and 4.3, respectively. Parathyroid auto-fluorescence prior to methylene blue injection was commonly observed.

**Conclusions:**

A clinical protocol for detection of fluorescence from MB during thyroid and parathyroid surgery is presented. Parathyroids (especially enlarged glands) fluoresce more intensely than thyroid glands. Auto-fluorescence may aid parathyroid detection, but MB fluorescence is needed to demonstrate viability.

## Introduction

Transient and long-term post-surgical hypoparathyroidism due to inadvertent removal, damage or devascularisation of parathyroid glands remains a common problem [1–4]. Accurate identification and differentiation between normal and enlarged parathyroid glands is essential for a good outcome.

Parathyroid glands can have a varied position and their small size and soft consistency increases the risk of inaccurate identification and damage at surgery. These glands may also be mistaken for other soft tissues such as lymph nodes or thyroid nodules, or vice versa.

Near-infrared fluorescence (NIRF) is one of several novel technologies that may be useful in early identification and preservation of parathyroid glands during surgery. Fluorophores re-emit light of a higher wavelength when excited by a light source. Some emit light outside of the visible spectrum in the near infra-red region (700–900 nm) [5]. NIRF has been used in surgery to aid real-time intraoperative visualisation of tissues and differentiate between tissue types [6–10] including sentinel lymph node mapping [6, 10–12]. Fluorophores that are currently available for clinical use include methylene blue (MB), indocyanine green (ICG) and 5-aminolevulinic acid (5-ALA). Intraoperative use of NIRF exploits the routes of metabolism and clearance of these agents in the body. ICG, excreted by the liver, is present in bile. This has been used to image bile ducts and liver cancers [8, 13–17]. MB, excreted via the kidneys, allows imaging of the ureters [7, 18]. Studies in neurosurgery have found that 5-ALA allows intraoperative assessment of tumours including meningioma [19].

Methylene blue is likely to be favourable for use in thyroid and/or parathyroid surgery as it is taken up readily by endocrine tissues [20, 21]. MB has traditionally been used intravenously in high doses (3–7.5 mg/kg) to aid naked eye identification of enlarged parathyroid glands by discolouring the gland blue. This method is thought to be very sensitive [22]. However, high-dose MB has many disadvantages. The operative field may be discoloured by blue dye; the staining may not be visualised through overlying tissue or fascia; there may be significant discolouration of the patient’s skin and urine and patients may suffer severe allergic reaction to the dye, but this is rare. At these high doses, MB also exerts neurotoxic effects, especially when used in conjunction with serotonin re-uptake inhibitors (SSRIs), adding significant morbidity to an otherwise relatively safe procedure [22, 23].

MB emits light in the near-infrared range (~ 700 nm). As near-infrared (NIR) light is invisible to the naked eye, the dye does not stain the operating field. Its presence is detected by a camera and is projected to a screen for visualisation. An additional advantage is that NIR radiation penetrates tissue better than visible light. The Fluobeam® 700 (Fluoptics, Grenoble, France) is a portable NIRF imaging system comprising of a control box linked to a camera head. The instrument head contains a class 1 laser (wavelength 680 nm), white light emitting diodes (LED) and a charge-coupled device camera. A high-band pass filter ensures that only fluorescent light over 700 nm is collected. The control box is linked to a laptop where real-time still and video images are displayed on screen and recorded.

We have previously investigated the use of MB and this imaging system in a rabbit model and showed that both thyroid and parathyroid glands fluoresced at low doses (0.025–3 mg/kg) of MB; the parathyroid glands showed a lower intensity of fluorescence with faster washout when compared to the thyroid gland [24].

A Phase Ia human study evaluated the feasibility of using NIRF imaging in the detection of parathyroid tissue and the patterns of fluorescent staining during parathyroidectomy [25]. This study showed that MB fluorescence can be demonstrated during surgery and there are differences in fluorescence between thyroid, parathyroid and other soft tissue structures. Although MB fluorescence from human thyroid and parathyroid tissue has been reported recently [26, 27], a systematic evaluation of dose response and temporal changes in fluorescence has not been performed.

The aim of the current study was to develop a protocol for the use of Fluobeam® 700 in combination with intravenous MB, for the detection of parathyroid tissue with differentiation from adjacent soft tissue, during thyroid and parathyroid surgery. The objectives were to identify the optimum dose of intravenous MB and time to peak fluorescence of normal and abnormal thyroid and parathyroid glands and to determine differences in patterns (onset, intensity and duration) of fluorescent staining between the various soft tissues of interest in the neck.

## Methods

This is an interventional study, without a control arm. Two groups of patients were included—patients with primary hyperparathyroidism (PHPT) undergoing parathyroidectomy (bilateral neck exploration/targeted parathyroidectomy) and patients undergoing thyroid resection (hemithyroidectomy/total thyroidectomy).

At thyroid and/or parathyroid surgery under general anaesthesia, the intervention was paused after exposure of the thyroid gland and at least one parathyroid gland to assess fluorescence. Accurate identification of the parathyroid gland relied on the surgeon’s judgement based on the appearance and location of the gland. In patients undergoing parathyroid surgery, the abnormal gland was identified. If the patient was undergoing a bilateral procedure, only one side of the neck was included in the recordings and assessments. The study could only proceed after at least one parathyroid gland was identified with confidence by the surgeon. At this stage, the Fluobeam® 700 camera in a sterile, transparent cover and connected to a laptop computer was held at a working distance of around 20 cm from the surgical field. The ambient room light was dimmed to a level similar to that used during laparoscopic surgery and the camera set at an exposure time of 83 ms. Intravenous methylene blue was then administered at a pre-determined dose based on results from previous participants in the study. For the first patient, a dose of 0.05 mg/kg body weight of MB was administered based on extrapolation from the previous animal experiment [24]. A sequence of images was recorded with a fixed interval time of 1 s between images. Recording was started before methylene blue was infused and continued for 10 min in the majority of patients. Continuous ECG and saturation monitoring was used in all patients. Blood pressure was monitored at 5-min intervals throughout the operation.

The study was divided into three stages—‘training’, ‘testing’ and ‘final’. The aim of the training stage was to estimate the optimal dose of MB, the duration of observation and to develop a working protocol. The dose of MB was refined in the testing stage before moving onto the final stage where the agreed protocol was tested. In the training and testing stages, MB doses starting at 0.05 mg/kg were administered and increased in increments of 0.05 mg/kg up to a maximum of 0.5 mg/kg in subsequent patients. The incremental change in dose depended on the results obtained from previous patients in the trial and the nature of the surgery (thyroid vs parathyroid) and was decided upon by the primary investigators (SH and SPB).

Images captured were analysed using ImageJ software [28]. Fluorescence intensity readings were taken from the thyroid gland, parathyroid gland, soft tissue (muscle or subcutaneous tissue) and surgical drapes. The mean grey scale measurement from the defined area was plotted against time. Ratios were calculated of the thyroid and parathyroid glands compared to soft tissue and the drapes and also plotted against time.

There are no formal statistical grounds (e.g. precision or power) on which an informed decision could be made regarding sample size calculations. A sample size of 50 (25 of each procedure) was selected on pragmatic grounds, keeping in mind the design of the protocol.

Onset of fluorescence and peak fluorescence readings were taken from the first 120 s after administration of MB. This captured the onset and first peak of fluorescence in all cases. As recording continued past 5 min, there was a significant increase in artefact from repositioning of hands and instruments and camera movement. Further peaks after this time were therefore disregarded. Analyses of fluorescent intensity were limited to the first 120 s of recording in patients given the final dose of 0.4 mg/kg MB. The first few seconds of recording, prior to administration of MB, was used to evaluate parathyroid auto-fluorescence.

Data analyses were primarily descriptive. The time to peak fluorescence of the thyroid and parathyroid glands and the fluorescent ratios were compared using the Wilcoxon signed rank test.

The study was approved by the Medicines and Healthcare Regulatory Agency (MHRA) (Reference number: 21304/0252/001-0001) and the Regional Research Ethics Committee (REC) (Reference number: 14/NW/0270). The study protocol was registered with ClinicalTrials.gov [29]. All patients included in the study were fully informed and gave written consent.

## Results

**Fig. 1:**
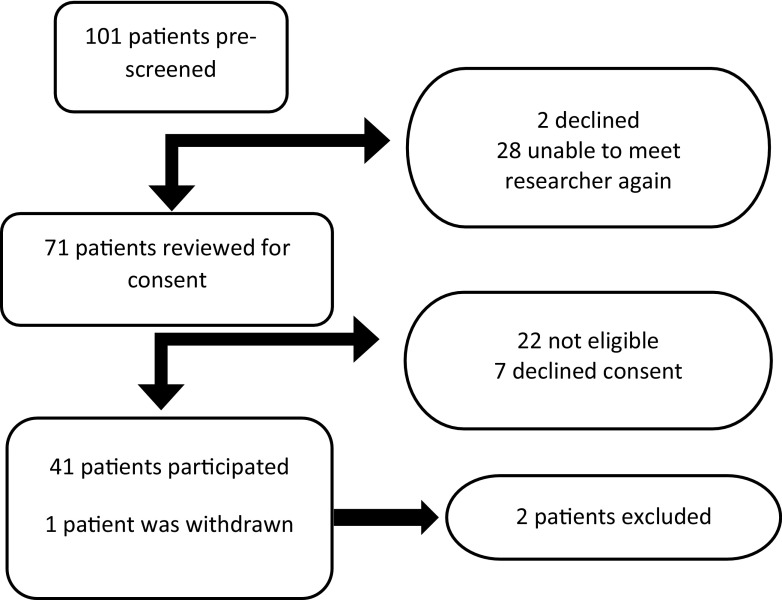
Recruitment flow chart

A total of 101 patients who were due to undergo thyroid and/or parathyroid surgery were screened for eligibility, approached for participation and given study information leaflets. A second meeting was planned with all patients to confirm eligibility and consent. The flow of patients from initial screening to inclusion in the study is shown in Fig. 1.

Of the 42 patients (36 female, 6 male), 32 underwent surgery for thyroid pathology and 10 for primary hyperparathyroidism. The median age (range) of participants was 49 (20–85). One patient was withdrawn prior to administration of MB as a decision was made not to interrupt the surgery due to anaesthetic difficulties that were encountered intraoperatively and is therefore not included in the study. Twenty-one patients were included in the training stage, 14 in the testing stage and 6 in the final stage. The training stage was used to find the approximate dose of MB that would be required by systematically increasing the dose by 0.05 mg/kg until fluorescence was reliably demonstrable in the tissues. The testing phase narrowed down the dose from a narrow range (0.3–0.5 mg/kg) to a single dose that was taken forward to the final stage (0.4 mg/kg). Two patients’ results were excluded as the laser was inadvertently switched off during recording in one and bright ambient light adversely affected detection of fluorescence in another.

There was a predictable, transient and spurious fall in oxygen saturation (pseudohypoxia) within the first minute of administration of MB, which recovered completely in all patients within 30 s. Oxygen saturations were shown to fall briefly to as low as 65%. All other hemodynamic parameters were stable at this time. This fall in saturation is due to an artefactual change in light absorbance as the dye in plasma interferes with photometric measurement of blood oxygen saturation [30, 31]. No patients had significant hypotension or ECG changes in relation to the administration of MB. No visible staining was observed of the glands or the rest of the surgical field. Postoperatively, some patients commented on a transient discolouration of urine but no other side effects were noted.

The parathyroid glands were often noted to be fluorescent and distinct from surrounding soft tissue prior to administration of methylene blue (auto-fluorescence). The mean difference in relative intensity between the PG and thyroid gland was +1.3 (*n* = 14) in the 10 s prior to MB administration in patients administered 0.4 mg/kg. Fluorescence was detected from the thyroid and PGs at doses as low as 0.05 mg/kg. However, at this dose, fluorescence was short-lived (around 8 s) and therefore unlikely to be useful in clinical practice.

Enlarged parathyroid glands (adenomatous or hyperplastic tissue in the context of primary hyperparathyroidism) showed increased fluorescence compared to normal glands. In one patient where an abnormal and normal parathyroid gland were both in view; at a dose of 0.1 mg/kg MB, the enlarged parathyroid gland demonstrated a peak fluorescence parathyroid: muscle ratio of 4.5 compared to 2.1 for the normal appearing gland. With increasing dose of MB, enlarged parathyroid glands showed intense fluorescence compared to surrounding tissues and in comparison to thyroid and normal PGs. Eleven PGs thought to be abnormal by the surgeon were sent for histology. All of these were shown on histology to be parathyroid tissue. Ten of these glands were abnormal (8 adenoma, 2 hyperplastic) and one was a normal parathyroid.

**Fig. 2:**
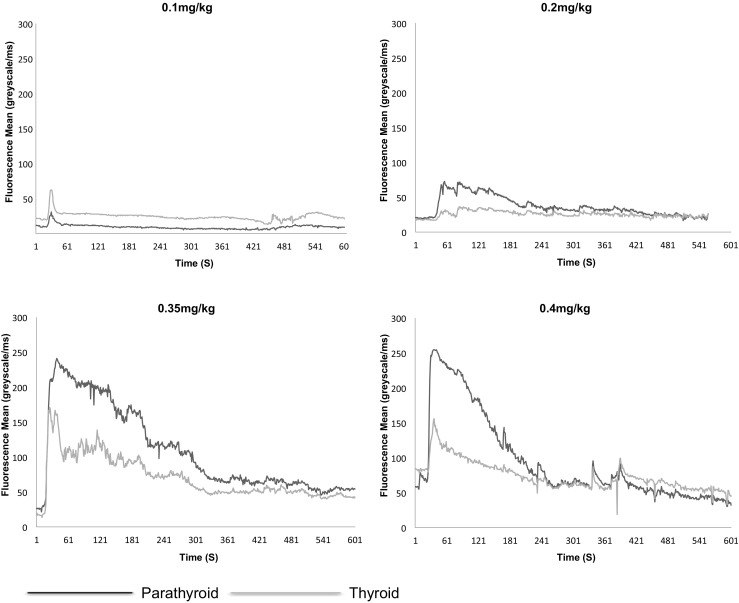
Mean fluorescence of normal parathyroid and abnormal thyroid glands over at four different concentrations of MB in individuals with Graves’ disease

**Fig. 3:**
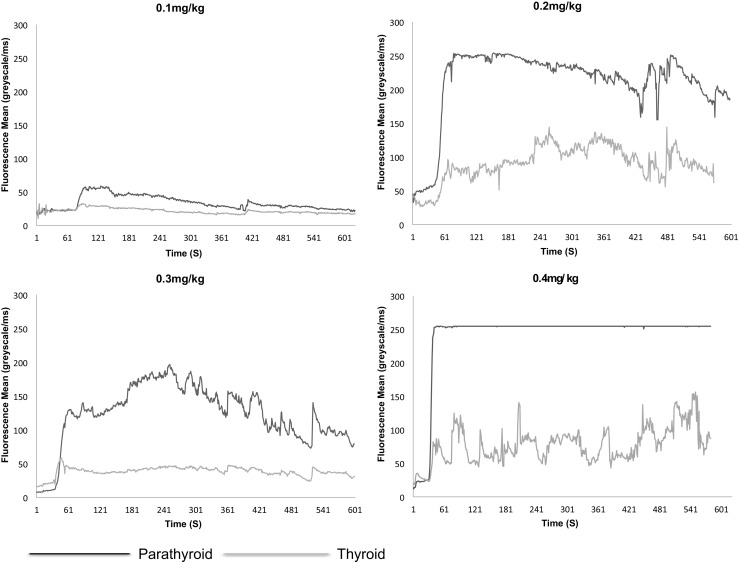
Mean fluorescence of abnormal parathyroid and normal thyroid glands over time at four different concentrations of MB in individuals with primary hyperparathyroidism

At the end of the testing phase, a dose of 0.4 mg/kg was adopted as the optimum dose (Figs. 2 and 3). At this dose, fluorescence from normal PGs was deemed to be reliable. A total of 15 patients received 0.4 mg/kg of MB (thyroid = 12, parathyroid = 3). The median of the times to onset of fluorescence, time to peak fluorescence, gland to ‘drape’ ratios and gland to muscle ratios at this dose are shown in Table 1.

**Table 1:** Fluorescent patterns of thyroid and parathyroid glands at dose of 0.4 mg/kg body weight

	Median time in seconds to onset of fluorescence (range)	Median time in seconds to peak fluorescence (range)	Median peak gland to drape ratio (range)	Median peak gland to muscle ratio (range)
Parathyroid gland (*n* = 15)	22.0 (13–33)	40.0 (26–88)	17.8 (6.7–54.8)	4.3 (1.7–6.8)
Thyroid gland (*n* = 14)	23.0 (12–34)	41.5 (27–103)	10.4 (5.7–29.7)	2.6 (1.1–5.1)

At this dose, the thyroid and PGs show a similar time to onset of fluorescence (*p* = 0.109) regardless of pathology. The time to peak fluorescence was 41.5 s (28–103) and 40s (26–88) for thyroid and parathyroid glands respectively and this was not significantly different (*p* = 0.859). PGs fluoresce more intensely than the thyroid when comparing either the gland to drape (*p* = 0.019) or gland to muscle ratios (*p* = 0.013). Abnormal parathyroid glands appear to fluoresce brighter than normal PGs, but the numbers within these sub groups are too small to test for statistical significance.

## Discussion

To our knowledge, this is the first human, phase I intraoperative dose response study of any fluorescent agent in surgical literature. The dose of 0.4 mg/kg MB appears to be the optimum level at which both thyroid and PGs reliably fluoresce for a clinically useful period of time. Increasing the dose does not improve the quality of images. At this dose, PGs appeared to fluoresce brighter than the adjacent thyroid tissue in the majority of patients. PG adenomas showed significantly brighter fluorescence compared to normal glands at the same dose.

**Fig. 4:**
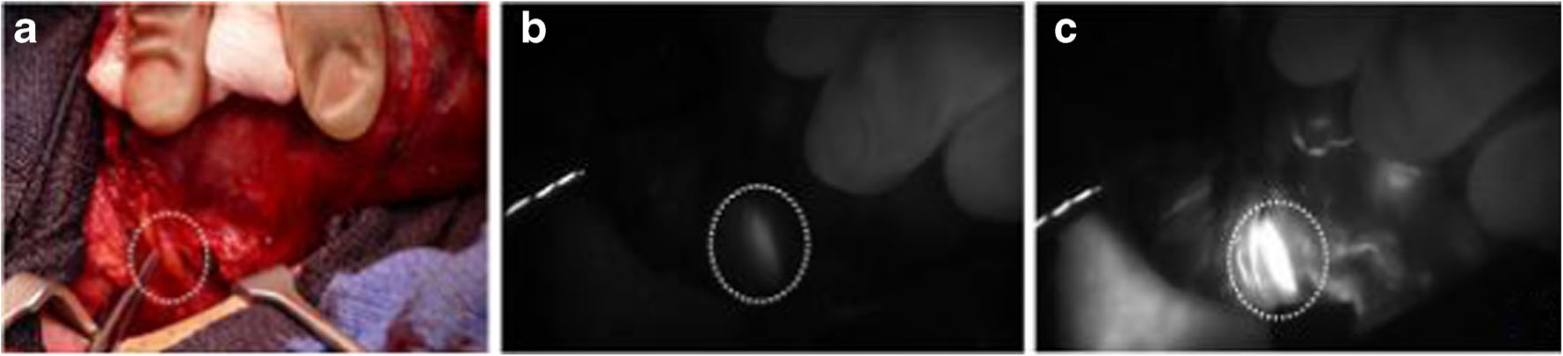
Patient with thyroid nodule undergoing right hemithyroidectomy. **a** Colour photograph of surgical field. Thyroid retracted medially by surgeon’s fingers. Normal superior PG identified dotted circle. **b** NIRF image prior to injection of MB showing parathyroid auto-fluorescence (dotted circle). **c** NIRF image at peak parathyroid fluorescence (42 s after injection of 0.4 mg/kg MB) showing significant PG fluorescence (dotted circle) compared to thyroid and surround soft tissues

Parathyroid glands have been shown to auto-fluoresce in the near-infrared spectrum. Two other studies evaluating parathyroid auto-fluorescence at wavelengths 750 nm [32] and 785 nm [33, 34] have shown that the parathyroid fluoresces between 2 and 11 times more than the thyroid or other neck structures [32–34]. The mechanism behind parathyroid auto-fluorescence is not yet understood and the fluorescence appears to be independent of pathology and gland viability [32, 35]. This study has demonstrated auto-fluorescence at excitation wavelength of 680 nm and this has not previously been observed. Although auto-fluorescence may aid intraoperative parathyroid identification, a further assessment of viability is needed to help make the decision for auto-transplantation as auto-fluorescence appears to be independent of perfusion. This is highlighted by an example in this study where there were concerns about viability in one parathyroid. Here, the PG was visible with use of the Fluobeam® 700 prior to the bolus of MB. The PG did not show any change in fluorescence after the bolus for up to 7 min after injection; which was not in keeping with other glands that were deemed to be viable. The demonstration of auto-fluorescence at 680 nm that is also compatible with MB fluorescence is of significance because the same device/technology can be used to both screen for the presence of parathyroid glands and also determine their viability after MB administration (Fig. 4).

Alternative imaging systems such as FLARE™ and Mini-FLARE™ have been used in some studies to assess MB fluorescence of parathyroid adenomas [26, 27]. A fixed dose of 0.5 mg/kg was used in all patients included in the studies, which concluded that parathyroid adenomas could be localised intraoperatively using this method and that normal PGs could occasionally be seen [26]. However, the basis of this fixed dose is not clear and no dose response assessments were included in the published reports.

Our study has shown that both normal and abnormal PGs can be identified at doses as low as 0.4 mg/kg (around 7.5–17.5 times lower than doses previously used for macroscopic blue staining). At this dose, the viability of a parathyroid gland can be confirmed and it may be possible to differentiate parathyroid adenoma from normal glands during a neck exploration. MB fluorescence can also be used to differentiate between PG and surrounding soft tissue such as lobules of brown fat or lymph nodes. Although the thyroid gland fluoresces following MB administration, the differences in auto-fluorescence along with MB fluorescence patterns assist in distinguishing parathyroids from thyroid tissue.

This study shows the potential for parathyroid auto-fluorescence at 680 nm to be used as a screening method for early parathyroid identification and MB fluorescence following intravenous administration for confirmation of parathyroid glands and assessment of its viability. The technology may be of help in a number of different scenarios—early parathyroid identification to enable preservation of normal glands during thyroidectomy; assessment of viability of parathyroid glands at the end of thyroidectomy to determine the need for auto-transplantation; and early identification of enlarged parathyroid glands in parathyroid surgery. This has in turn the potential to improve outcomes of thyroid and parathyroid surgery. As the onset of fluorescence from the tissues is seen within seconds of MB administration, the presented method enables a rapid assessment of viability without extensive preparation.

There are a number of factors that affect the quantitative assessment of fluorescent intensity during the operation. These include the distance of the camera from the tissues due to operator use or depth of the surgical field, the position of the gland within the field of view and degradation of the image due to the original sterile cover. Adjusting for all these factors and keeping all extraneous factors constant is difficult in clinical practice. However, despite these extraneous influences, an objective assessment of fluorescent intensity using ImageJ enabled comparison of fluorescence between soft tissues at different doses and over time. This has resulted in the development of a clinical protocol that can be used in a pragmatic manner in the clinical setting. For the purpose of the experiments, both the thyroid and PG remained in a fixed field of view throughout the recording. This may affect the fluorescence detected by the camera from the more peripheral areas of the field of view compared to the centre as the laser excitation is highest in the centre. In practice, surgeons may use the camera in a less static manner and therefore the centre of the field of view may change as different areas of the neck are investigated. ImageJ was used post-acquisition as a method of quantifying the fluorescence detected from the images obtained. A mean greyscale measurement of the pixels within a chosen area is a representation of fluorescence. The areas of interest were determined by free-hand selection. The images were occasionally degraded by surgical instruments or hands entering the field of view and obscuring all or part of a gland. In this case, an area was selected which best represented the gland. It was also noted that over the period of 10 min there was operator fatigue and movement of the tissues of interest outside the field of view. During the repositioning of the instruments or surgeon’s fingers used for retraction of the thyroid, there were changes in fluorescence due to changes in exposure, movement from the centre of the field of view or changes in blood supply due to pressure on adjacent vasculature. Whilst attempts were made to take the reading from muscle, some of these readings may have been at least in part from adjacent subcutaneous soft tissue as it was difficult to always ensure that the strap muscles were in view during recording.

The MB was injected via a tubing connected to a peripheral cannula. The size of this tubing varied in diameter, depending on the anaesthetist’s preference. It was noted that despite flushing of the tubing after administration of MB, occasionally, MB was left in the tubing. It is therefore recommended that MB is administered via a fine tube or connector to the peripheral cannula.

In conclusion, parathyroid glands can be identified intraoperatively using NIR imaging following administration of intravenous methylene blue at doses as low as 0.4 mg/kg. PG fluorescence is observed within 60 s of administration and PGs fluoresce more intensely than the thyroid tissue. Enlarged parathyroid glands may be distinguished from normal glands based on the fluorescence intensity. This method has potential in enabling early and accurate identification of parathyroid glands and determining gland viability, thereby improving outcomes in thyroid and parathyroid surgery. A phase II study of this protocol to evaluate clinical outcomes such as post thyroidectomy hypoparathyroidism is now required.

## References

[1] Antakia R, Edafe O, Uttley L, Balasubramanian SP (2015). Effectiveness of preventative and other surgical measures on hypocalcemia following bilateral thyroid surgery: a systematic review and meta-analysis. Thyroid.

[2] Chadwick D, Kinsman R, Walton P (2012) The British Association of Endocrine and Thyroid Surgeon Fourth National Audit. Dendrite Clinical Systems Ltd, Henley-on-Thames. Available from: http://www.baets.org.uk/Pages/4th%20National%20Audit.pdf

[3] Steen S, Rabeler B, Fisher T, Arnold D (2009). Predictive factors for early postoperative hypocalcemia after surgery for primary hyperparathyroidism. Proc (Bayl Univ Med Cent).

[4] Basheeth N, O'Cathain E, O'Leary G, Sheahan P (2014). Hypocalcemia after total laryngectomy: incidence and risk factors. Laryngoscope.

[5] Schaafsma BE, Mieog JS, Hutteman M, van der Vorst JR, Kuppen PJ, Lowik CW (2011). The clinical use of indocyanine green as a near-infrared fluorescent contrast agent for image-guided oncologic surgery. J Surg Oncol.

[6] Crane LM, Themelis G, Arts HJ, Buddingh KT, Brouwers AH, Ntziachristos V (2011). Intraoperative near-infrared fluorescence imaging for sentinel lymph node detection in vulvar cancer: first clinical results. Gynecol Oncol.

[7] Matsui A, Tanaka E, Choi HS, Kianzad V, Gioux S, Lomnes SJ, Frangioni JV (2010). Real-time, near-infrared, fluorescence-guided identification of the ureters using methylene blue. Surgery.

[8] Matsui A, Tanaka E, Choi HS, Winer JH, Kianzad V, Gioux S, Laurence RG, Frangioni JV (2010). Real-time intra-operative near-infrared fluorescence identification of the extrahepatic bile ducts using clinically available contrast agents. Surgery.

[9] Tummers QR, Boonstra MC, Frangioni JV, van de Velde CJ, Vahrmeijer AL, Bonsing BA (2015). Intraoperative near-infrared fluorescence imaging of a paraganglioma using methylene blue: a case report. Int J Surg Case Rep.

[10] van der Vorst JR, Schaafsma BE, Verbeek FP, Keereweer S, Jansen JC, van der Velden LA (2013). Near-infrared fluorescence sentinel lymph node mapping of the oral cavity in head and neck cancer patients. Oral Oncol.

[11] Hutteman M, van der Vorst JR, Gaarenstroom KN, Peters AA, Mieog JS, Schaafsma BE (2012). Optimization of near-infrared fluorescent sentinel lymph node mapping for vulvar cancer. Am J Obstet Gynecol.

[12] van der Vorst JR, Schaafsma BE, Verbeek FP, Swijnenburg RJ, Hutteman M, Liefers GJ (2013). Dose optimization for near-infrared fluorescence sentinel lymph node mapping in patients with melanoma. Br J Dermatol.

[13] Gotoh K, Yamada T, Ishikawa O, Takahashi H, Eguchi H, Yano M, Ohigashi H, Tomita Y, Miyamoto Y, Imaoka S (2009). A novel image-guided surgery of hepatocellular carcinoma by indocyanine green fluorescence imaging navigation. J Surg Oncol.

[14] Hutteman M, van der Vorst JR, Mieog JS, Bonsing BA, Hartgrink HH, Kuppen PJ (2011). Near-infrared fluorescence imaging in patients undergoing pancreaticoduodenectomy. Eur Surg Res.

[15] Ishizawa T, Fukushima N, Shibahara J, Masuda K, Tamura S, Aoki T, Hasegawa K, Beck Y, Fukayama M, Kokudo N (2009). Real-time identification of liver cancers by using indocyanine green fluorescent imaging. Cancer.

[16] Lim C, Vibert E, Azoulay D, Salloum C, Ishizawa T, Yoshioka R, Mise Y, Sakamoto Y, Aoki T, Sugawara Y, Hasegawa K, Kokudo N (2014). Indocyanine green fluorescence imaging in the surgical management of liver cancers: current facts and future implications. J Visc Surg.

[17] Sakoda M, Ueno S, Iino S, Hiwatashi K, Minami K, Kawasaki Y, Kurahara H, Mataki Y, Maemura K, Uenosono Y, Shinchi H, Natsugoe S (2014). Anatomical laparoscopic hepatectomy for hepatocellular carcinoma using indocyanine green fluorescence imaging. J Laparoendosc Adv Surg Tech A.

[18] Hsu M, Gupta M, Su LM, Liao JC (2014). Intraoperative optical imaging and tissue interrogation during urologic surgery. Curr Opin Urol.

[19] Vahrmeijer AL, Hutteman M, van der Vorst JR, van de Velde CJ, Frangioni JV (2013). Image-guided cancer surgery using near-infrared fluorescence. Nature reviews. Clin Oncol.

[20] Hurvitz RJ, Perzik SL, Morgenstern L (1968). In vivo staining of the parathyroid glands. A clinical study. Arch Surg.

[21] Dudley NE (1971). Methylene blue for rapid identification of the parathyroids. Br Med J.

[22] Patel HP, Chadwick DR, Harrison BJ, Balasubramanian SP (2012). Systematic review of intravenous methylene blue in parathyroid surgery. Br J Surg.

[23] Vutskits L, Briner A, Klauser P, Gascon E, Dayer AG, Kiss JZ, Muller D, Licker MJ, Morel DR (2008). Adverse effects of methylene blue on the central nervous system. Anesthesiology.

[24] Antakia R, Gayet P, Guillermet S, Stephenson TJ, Brown NJ, Harrison BJ, Balasubramanian SP (2014). Near infrared fluorescence imaging of rabbit thyroid and parathyroid glands. J Surg Res.

[25] ClinicalTrials.gov (2012) Near Infrared Fluorescent Imaging in Thyroid and Parathyroid Surgery With the Fluobeam(TM) System of Fluoptics. Available from: https://clinicaltrials.gov/ct2/show/NCT01598727?term=fluorescence+parathyroid&rank=2

[26] Tummers QR, Schepers A, Hamming JF, Kievit J, Frangioni JV, van de Velde CJ (2015). Intraoperative guidance in parathyroid surgery using near-infrared fluorescence imaging and low-dose methylene blue. Surgery.

[27] van der Vorst JR, Schaafsma BE, Verbeek FP, Swijnenburg RJ, Tummers QR, Hutteman M (2014). Intraoperative near-infrared fluorescence imaging of parathyroid adenomas with use of low-dose methylene blue. Head Neck.

[28] Rasband WS (1997–2016) ImageJ Bethesda, Maryland, USA: U. S. National Institutes of Health. Available from: http://imagej.nih.gov/ij/

[29] ClinicalTrials.gov (2014) Intra-operative Infra-red Fluorescent Imaging in Thyroid and Parathyroid Surgery. Available from: https://clinicaltrials.gov/ct2/show/NCT02089542?term=balasubramanian&rank=1

[30] Sidi A, Paulus DA, Rush W, Gravenstein N, Davis RF (1987). Methylene blue and indocyanine green artifactually lower pulse oximetry readings of oxygen saturation. Studies in dogs. J Clin Monit.

[31] Varon AJ, Anderson HB, Civetta JM (1989). Desaturation noted by pulmonary artery catheter oximeter after methylene blue injection. Anesthesiology.

[32] De Leeuw F, Breuskin I, Abbaci M, Casiraghi O, Mirghani H, Ben Lakhdar A, Laplace-Builhé C, Hartl D (2016). Intraoperative near-infrared imaging for parathyroid gland identification by auto-fluorescence: a feasibility study. World J Surg.

[33] McWade MA, Paras C, White LM, Phay JE, Solorzano CC, Broome JT (2014). Label-free intraoperative parathyroid localization with near-infrared autofluorescence imaging. J Clin Endocrinol Metab.

[34] Paras C, Keller M, White L, Phay J, Mahadevan-Jansen A (2011). Near-infrared autofluorescence for the detection of parathyroid glands. J Biomed Opt.

[35] McWade MA, Paras C, White LM, Phay JE, Mahadevan-Jansen A, Broome JT (2013). A novel optical approach to intraoperative detection of parathyroid glands. Surgery.

